# Selective anticancer agents suppress aging in *Drosophila*

**DOI:** 10.18632/oncotarget.1272

**Published:** 2013-09-07

**Authors:** Anton Danilov, Mikhail Shaposhnikov, Ekaterina Plyusnina, Valeria Kogan, Peter Fedichev, Alexey Moskalev

**Affiliations:** ^1^ Institute of Biology, Komi Science Center, Russian Academy of Sciences, Syktyvkar, 167982, Russia; ^2^ Syktyvkar State University, Syktyvkar, 167001, Russia; ^3^ Moscow Institute of Physics and Technology, Dolgoprudny, Moscow Region, 141700, Russia; ^4^ Quantum Pharmaceuticals, Ul. Kosmonavta Volkova 6A-1205, Moscow, 125171, Russia

**Keywords:** longevity, quality of life, aging-suppressive substances, anticancer agents, PI3K, TOR, iNOS, NF-κB, Drosophila melanogaster

## Abstract

Mutations of the *PI3K*, *TOR*, *iNOS*, and *NF-κB* genes increase lifespan of model organisms and reduce the risk of some aging-associated diseases. We studied the effects of inhibitors of PI3K (wortmannin), TOR (rapamycin), iNOS (1400W), NF-κB (pyrrolidin dithiocarbamate and QNZ), and the combined effects of inhibitors: PI3K (wortmannin) and TOR (rapamycin), NF-κB (pyrrolidin dithiocarbamates) and PI3K (wortmannin), NF-κB (pyrrolidine dithiocarbamates) and TOR (rapamycin) on *Drosophila melanogaster* lifespan and quality of life (locomotor activity and fertility). Our data demonstrate that pharmacological inhibition of PI3K, TOR, NF-κB, and iNOS increases lifespan of *Drosophila* without decreasing quality of life. The greatest lifespan expanding effect was achieved by a combination of rapamycin (5 μM) and wortmannin (5 μM) (by 23.4%). The bioinformatic analysis (KEGG, REACTOME.PATH, DOLite, and GO.BP) showed the greatest aging-suppressor activity of rapamycin, consistent with experimental data.

## INTRODUCTION

According to Build 16 of GenAge database (http://genomics.senescence.info/genes/), it is known more than 1700 genes associated with longevity in model organisms, and their number is constantly increasing [1]. Among the most known evolutionarily conserved longevity genes are *TOR* (target of rapamycin) [2-4], *PI3K* (phosphatidylinositide 3-kinase) [5], *NF-κB* (nuclear factor-kappaB) [6, 7] and *iNOS* (inducible nitric oxide synthase) [8, 9]. Therefore, we selected the products of these genes as targets for pharmacological inhibition. Indeed, pharmacological inhibition of the activity TOR [10-19], PI3K [12, 20, 21], NF-κB [22] and iNOS [23] increases the lifespan in yeasts, worms, flies and mammals.

However despite the significant progress the effects of low concentrations of inhibitors of longevity genes products, sex-specific effects, and combination effects of different inhibitors remain unclear. We suggest that application of compounds in low concentrations may reduce the risk of side effects. Another problem is that drugs may demonstrate gender-specific efficiencies and sex-dependent side effects [24, 25]. The aging is a complex process that involves many intracellular signaling pathways, we made the assumption that the most pronounced effect on lifespan will have a combined inhibition of aging-associated signaling pathways.

The aging process is associated with hyperactivation of TOR and PI3K [26], as well as NF-κB [27] and iNOS [28, 29], leading to cellular senescence, age-related pathologies, and oncogenesis. Therefore, many anticancer agents are inhibitors of the same enzymes as aging-suppressors, including TOR [26, 30-32], PI3K [33], NF-κB [34] and iNOS [35]. This is entirely consistent with the theory that considers cellular senescence as age-dependent hyperactivation of pro-aging signaling pathways [26, 36].

Thus, the molecular mechanisms of aging and carcinogenesis are interrelated. In particular, long-living mammals, such as the naked mole rat, mole rat and the whale, have reduced cancer incidence [37-40]. Another long-living mammal – microbat *Myotis brandtii*, as we found out, has multiple extra copies of the onco-suppressor gene *FBXO31*, and probably also resistant to tumorigenesis [41]. Bioinformatic analysis of the relationship between longevity- and cancer-associated genes/proteins revealed remarkable trend from yeast to humans: tumor suppressors orthologs are associated with lifespan extension, whereas the proto-oncogenes orthologs are associated with reduced lifespan [42]. For example, pro-longevity function is appropriate for tumor suppressors FOXO3a [43], PTEN [44], p53 [45], however gerontogenes PI3K and mTOR promote oncogenesis [46, 47].

Most of the 9 age-related disorders (genomic instability, telomere attrition, epigenetic alterations, loss of proteostasis, deregulated nutrient sensing, mitochondrial dysfunction, cellular senescence, stem cell exhaustion, and altered intercellular communication), as well as one additional – chronic inflammation may be potentially corrected by pharmacological interventions [48].

Phenotypic screening of compounds that increase model organisms lifespan by regulating the activity of gerontogenes products and using of gerontogenes products as targets for molecular modeling techniques (computer-aided drug design) could be the first steps for the development of new drugs for treating aging-associated diseases, including various cancers [49].

The purpose of this study was to examine aging-suppressor properties of specific kinase inhibitors TOR (rapamycin), phosphoinositide 3-kinase (wortmannin) (PDTC and QNZ) and inducible NO synthase iNOS (1400W) on the lifespan, locomotor activity and fertility of *Drosophila melanogaster*.

## RESULTS

### Effects on the lifespan and life quality

In this paper, we conducted a comprehensive study of aging-suppressor properties of 5 inhibitors and their combinations in several nano and micro molar concentrations. The targets of studied inhibitors are the products of gerontogenes such as TOR, PI3K, NF-κB and iNOS.

**Figure 1:**
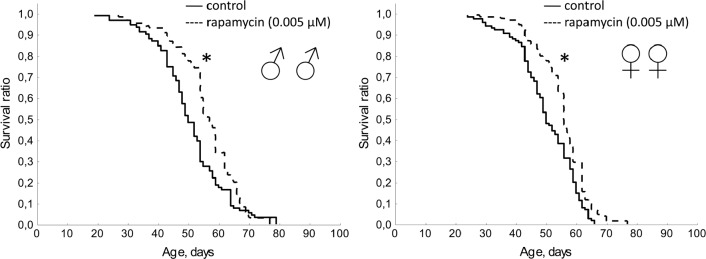
Effect of rapamycin (0.005 μM) on lifespan Drosophila melanogaster * p< 0.001, ** p< 0.05 (Kolmogorov-Smirnov test).

**Figure 2:**
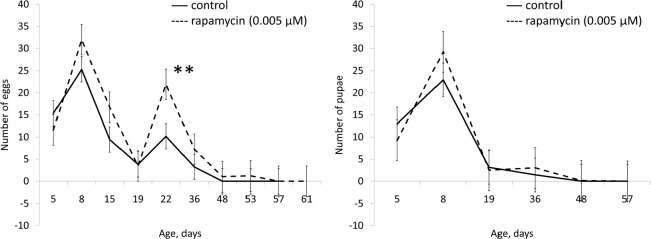
Effect of rapamycin (0.005 μM) on fertility of females Drosophila melanogaster * p< 0.001, ** p< 0.05 (χ^2^ test).

Exposure to rapamycin (0.005 μM) caused a statistically significant (p <0.01) increase in median lifespan in males (by 14%) and females (by 12%) (Table 1, Fig. 1). Rapamycin in concentration of 0.005 μM increased activity in negative geotaxis test in males, and significantly increased fertility of females (Table 1, Fig. 2 and S1).

**Table 1:** Effect of the TOR, PI3K and NF-κB inhibitors on the lifespan, fertility and locomotor activity Drosophila melanogaster

Compound (concentration)	Target	Lifespan						LA		F
		♂			♀			♂	♀	♀
		M	90%	n	M	90%	n			
Rapa (0.005 μM)	TOR	↑ (+14%)**	↑(+4.7%)	344	↑(+12%)**	↑(+4.8%)	363	↑	↑	=
Wm (0.005 μM)	PI3K	↑(+8%)	↑(+4.7%)	312	↑(+8%)	↑(+4.8%)	355	↑	=	=
Wm (5 μM)	PI3K	↑ (+5%)*	↑ (+2%)	259	↓ (-8.2%)*	↓ (-1.7%)	252	↑	=	=
PDTC (1.25 μM)	NF-κB	↑(+6%)*	↑(+2%)	437	↓(-2%)*	↓(-5%)*	451	↑	=	=
PDTC (12.5 μM)	NF-κB	↑(+7%)**	↑(+6%)**	448	↓(-2%)*	=	433	=	=	↑
PDTC (125 μM)	NF-κB	↑(+10%)*	↓(-3.1%)*	322	↑(+12%)*	↑(+4.8%)	361	=	=	=
QNZ (0.03 μM)	NF-κB	↑(+2%)	↑(+4%)	297	↓(-15%)**	↓(-4%)**	298	=	↑	↑
QNZ (0.3 μM)	NF-κB	=	↑(+8%)	279	↓(-4%)	↓(-4%)**	290	↓	=	↑
QNZ (3 μM)	NF-κB	↑(+2%)	=	282	↓(-4%)*	↓(-4%)**	312	↑	=	↑
1400W (0.03 μM)	iNOS	↑(+3%)**	↑(+13%)**	396	↓(-5%)**	↓(-5%)**	437	=	↓	↑
1400W (0.3 μM)	iNOS	=	=	419	↓(-2%)**	↓(-5%)**	453	↑	↑	↑
1400W (3 μM)	iNOS	↑(+7%)**	↑(+5%)*	428	↓(-2%)**	↓(-4%)**	449	↑	↓	=
Rapa (5 μM) + Wm (5 μM)	TOR + PI3K	↑ (+2.4%)*	↑ (+8%)	372	↑ (+14.6%)*	↑ (+23.4%)*	400	-	-	-
PDTC (125 μM) + Rapa (0.005 μM)	NF-κB + TOR	↑(+10%)**	↓(-3.1%)	305	↑(+10%)*	↑(+11.3%)**	370	↑	↑	=
PDTC (125 μM) + Wm (0.005 μM)	NF-κB + PI3K	↑(+10%)*	↑(+4.7%)	304	↑(+12%)*	↑(+8.1%)*	342	↑	↑	=

*Legend*: «↑» – increasing, «↓» - decreasing, "=" – no effect "-" – not studied, M - median lifespan, 90% - the age of 90% mortality, n - sample size, LA - locomotor activity, F – fertility, *p<0.05, ***p<0.01 – Gehan–Breslow–Wilcoxon and Mantel–Cox tests for M, Wang-Allison test for 90%.

**Figure 3:**
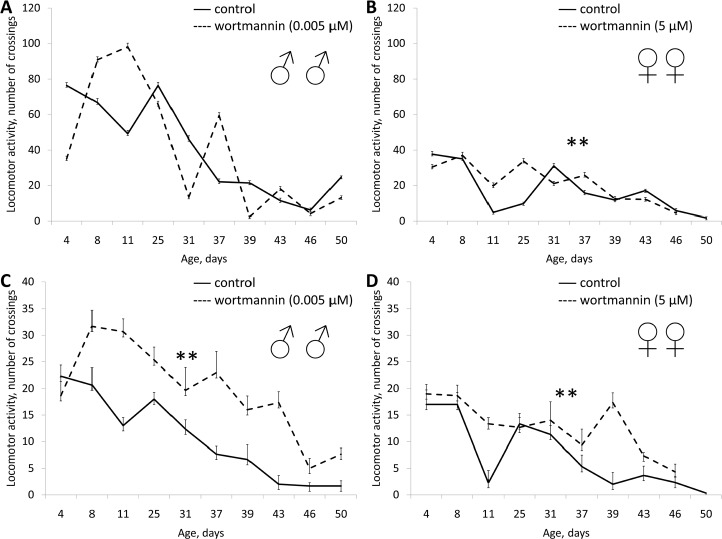
Effect of wortmannin (0.005 μM) on locomotor activity Drosophila melanogaster (A, B) spontaneous activity. (C, D) negative geotaksis test. * p< 0.001, ** p< 0.05 (χ^2^ test).

Wortmannin in concentration of 5 μM increased the median lifespan of males by 5% (p <0.05), but decreased in females by 8.2% (p <0.05). In concentration of 0.005 μM wortmannin had no statistically significant effect on lifespan (Table 1, Fig. S2). Treatment with wortmannin in a concentration of 5 μM decreased spontaneous locomotor activity in females during the second half of life, however, however the end of life activity in treated females was higher than in the control. Wortmannin in concentrations of 0.005 and 5 μM increased the reproductive period in females (Table 1, Fig. S3). Wortmannin in concentration of 0.005 μM and 5 μM in males and 5 μM in females significantly increased activity in the test on negative geotaxis (Table 1, Fig. 3 and S4).

**Figure 4:**
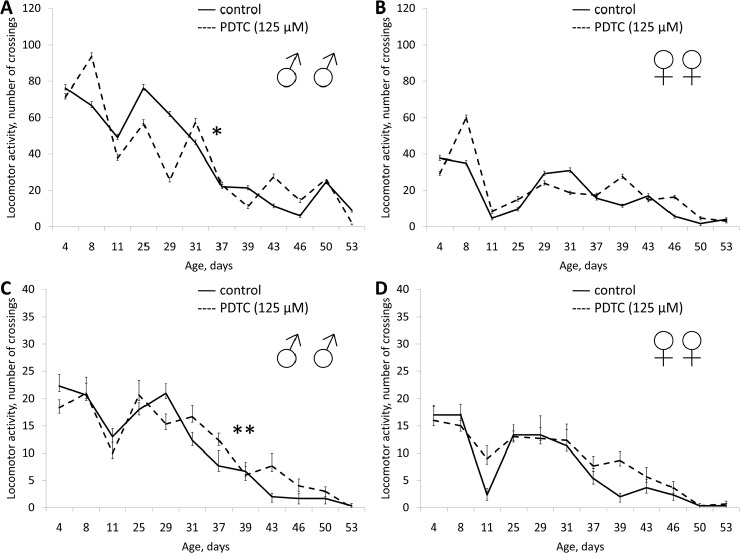
Effect of PDTC (125 μM) on locomotor activity Drosophila melanogaster (A, B) spontaneous activity. (C, D) negative geotaksis test. * p< 0.001, ** p< 0.05 (x^2^ test).

In males treatment with PDTC in concentrations of 1.25, 12.5 and 125 μM resulted in increasing of median lifespan by 6-10% (p<0.01) and increasing of the age of 90% mortality by 2 and 6 %, respectively (Table 1). In females PDTC in concentration of 125 μM increased median lifespan by 12% (Table 1, Fig. S5). PDTC in concentration of 12.5 μM increased female fertility (Table 1, Fig. S6). PDTC in a concentration of 1.25 μM increased the locomotor activity of males (Table 1, Fig. S7). However in concentrations of 12.5 and 125 μM PDTC did not affect the locomotor activity of males (Table 1, Fig. 4 and S8).

**Figure 5:**
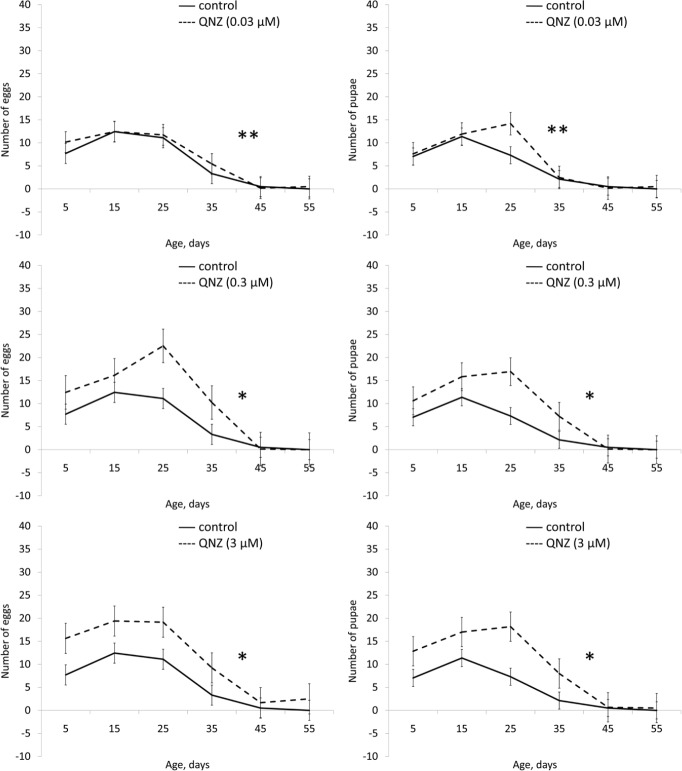
Effect of QNZ (0.03, 0.3, 3 μM) on fertility of females Drosophila melanogaster * p< 0.001, ** p< 0.05 (x^2^ test).

**Figure 6:**
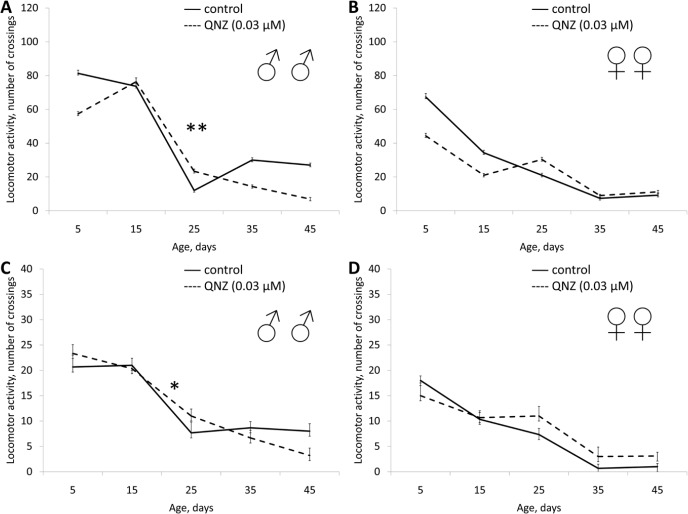
Effect of QNZ (0.03 μM) on locomotor activity Drosophila melanogaster (A, B) spontaneous activity. (C, D) negative geotaksis test. * p< 0.001, ** p< 0.05 (x^2^ test).

**Figure 7:**
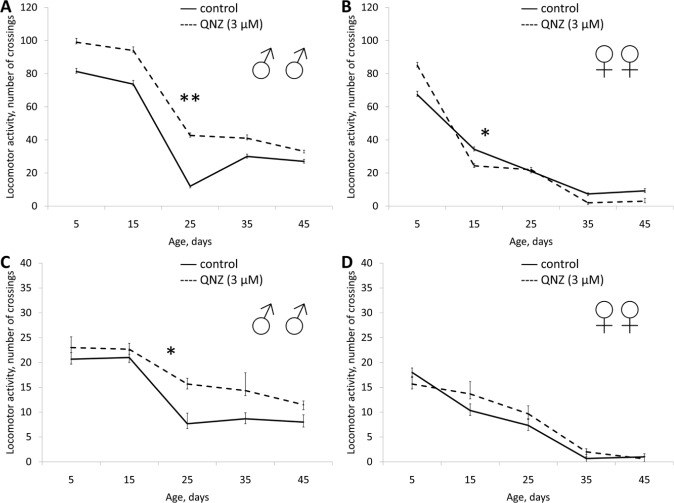
Effect of QNZ (3 μM) on locomotor activity Drosophila melanogaster (A, B) spontaneous activity. (C, D) negative geotaksis test. * p< 0.001, ** p< 0.05 (x^2^ test).

Application of QNZ did not affect the lifespan of males in all studied concentrations (3, 0.3, 0.03 μM). In females QNZ induced decreasing of lifespan (Table 1, Fig. S9). QNZ in concentrations of 3, 0.3, 0.03 μM increased female fecundity (Table 1, Fig. 5). QNZ in concentration of 0.03 μM increased the locomotor activity of females (Fig. 6) and in the concentration of 3 μM activity of the males (Fig. 7). Treatnent with QNZ in concentration of 0.3 μM did not affect activity of males or females (Fig. S10)

**Figure 8:**
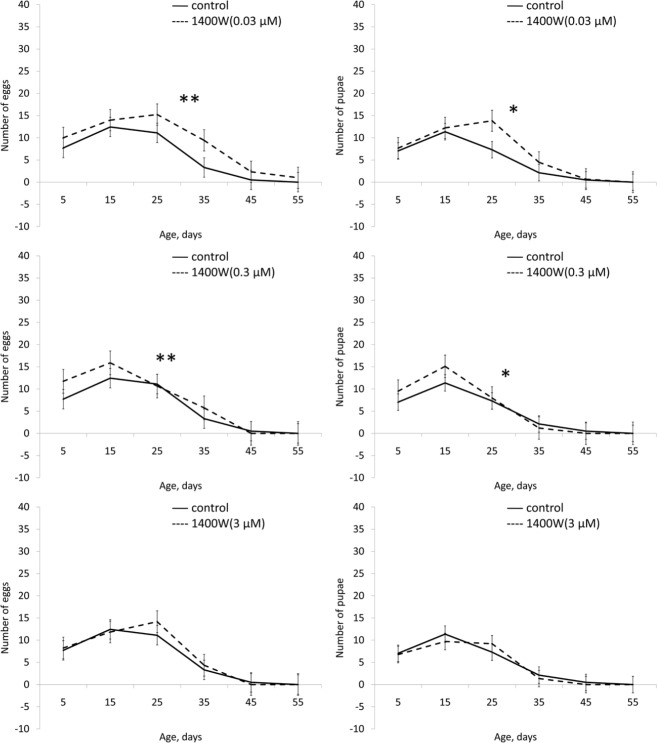
Effect of 1400W (0.3 μM) on locomotor activity Drosophila melanogaster (A, B) spontaneous activity. (C, D) negative geotaksis test. * p< 0.001, ** p< 0.05 (x^2^ test).

**Figure 9:**
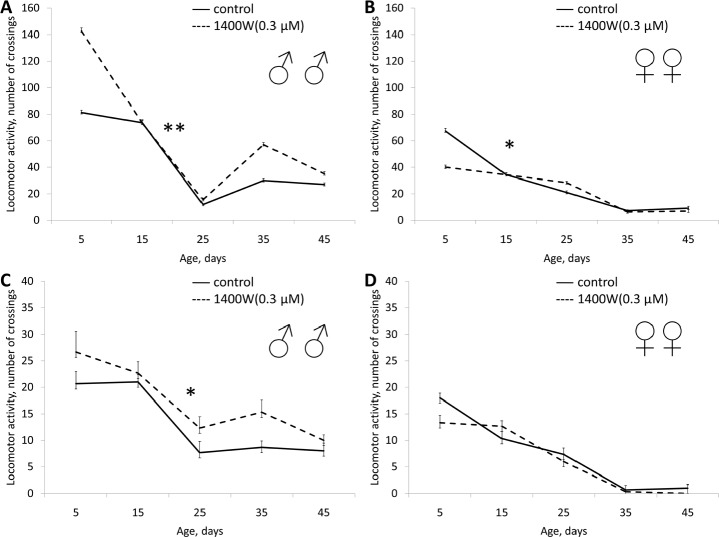
Effect of 1400W (0.03, 0.3, 3 μM) on fertility of females Drosophila melanogaster * p< 0.001, ** p< 0.05 (x^2^ test).

In males 1400W in concentrations of 0.03 and 3 μM and significantly increased the median lifespan (by 3 and 7%, respectively) and the age of 90% mortality (by 13 and 5%, respectively). In females we observed a decrease in median lifespan (by 2-5%) and the age of 90% mortality (by 4-5%) when exposed to 1400W at different concentrations (Table 1, Fig. S11). In concentrations of 0.3 and 3 0.03 μM 1400W increased the locomotor activity of the males (Fig. 8, S12 and S13). In females locomotor activity increased only when exposed to 1400W in concentration of 0.3 μM (Fig. 8, S12 and S13). 1400W in concentration of 0.3 μM increased female fertility during the first half of life, and in concentration of 0.03 μM increased fertility throughout life (Table 1, Fig. 9).

PI3-kinase, TOR and NF-κB transcription factor – are the elements of different intracellular signaling pathways the impact of which may increase the lifespan of model organisms. We hypothesized that the combined application of substances that inhibit different targets will lead to a greater increase in lifespan than the application of each agent substance.

We examined 2 types of mixtures with a high concentration of PDTC (125 μM) and low concentrations of wortmannin and rapamycin (0.005 μM) as well as mixture with the same concentrations of wortmannin and rapamycin (5 μM).

**Figure 10:**
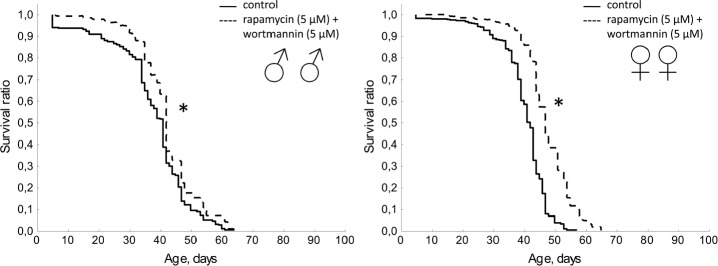
Effect of combined use of rapamycin (5 μM) and wortmannin (5 μM) on lifespan Drosophila melanogaster * p< 0.001, ** p< 0.05 (Kolmogorov-Smirnov test).

As a result of combined application of wortmannin (5 μM) and rapamycin (5 μM) we observed the increase in the median lifespan by 14.6%, and the age of 90% mortality by 23.4% in females. The median lifespan of males increased by 2.4% (Table 1, Fig. 10).

**Figure 11:**
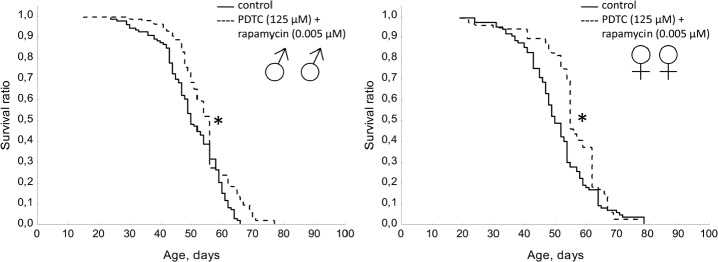
Effect of combined use of PDTC (125 μM) and rapamycin (0.005 μM) on lifespan Drosophila melanogaster * p< 0.001, ** p< 0.05 (Kolmogorov-Smirnov test).

**Figure 12:**
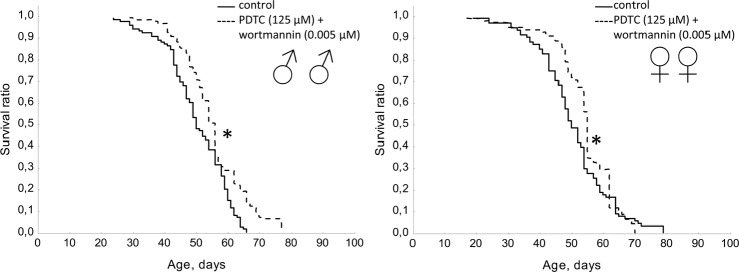
Effect of combined use of PDTC (125 μM) and wortmannin (0.005 μM) on lifespan Drosophila melanogaster * p< 0.001, ** p< 0.05 (Kolmogorov-Smirnov test).

**Figure 13:**
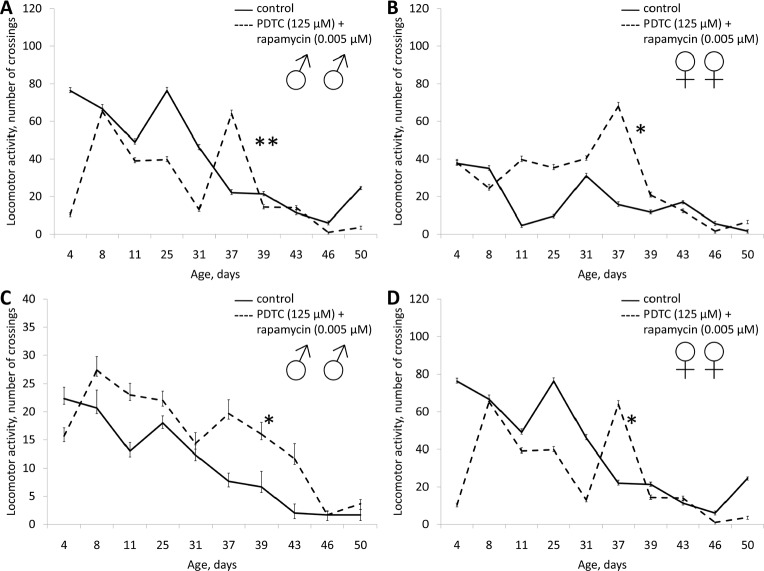
Effect of combined use of PDTC (125 μM) and rapamycin (0.005 μM) on locomotor activity Drosophila melanogaster (A, B) spontaneous activity. (C, D) negative geotaksis test. * p< 0.001, ** p< 0.05 (x^2^ test).

**Figure 14:**
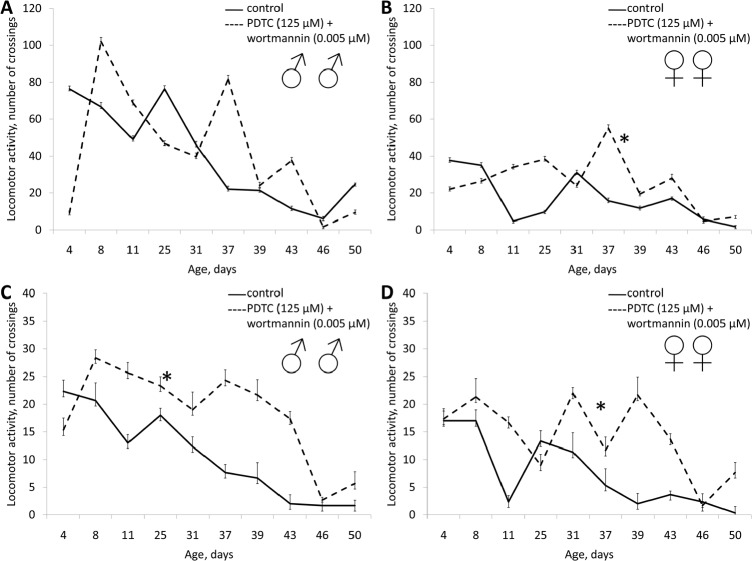
Effect of combined use of PDTC (125 μM) and wortmannin (0.005 μM) on locomotor activity Drosophila melanogaster (A, B) spontaneous activity. (C, D) negative geotaksis test. * p< 0.001, ** p< 0.05 (x^2^ test).

A mixture of PDTC (125 μM) with wortmannin (0.005 μM), and PDTC (125 μM) with rapamycin (0.005 μM) increased the median lifespan in males (by 10%) and females (by 10 and 12%, respectively). We also observed an increase in the age of 90% mortality in females by 11.3% and 8.1% respectively (Table 1, Fig. 11 and 12). The study of age-related dynamics of female fertility revealed no adverse effects of mixtures of PDTC (125 μM) with rapamycin (5 μM) and PDTC (125 μM) with wortmannin (5 μM) (Table 1, Fig. S14). When flies were exposed to mixtures of PDTC (125 μM) with wortmannin (5 μM) and PDTC (125 μM) with rapamycin (5 μM) we observed a significant increase in locomotor activity of males and females in the test on negative geotaxis and increasing of spontaneous activity in females (Table 1, Fig. 13 and 14).

### Bioinformatic analysis

Bioinformatic analysis of the target of rapamycin, wortmannin, PDTC and 1400W, retrieved from the ChEMBL database, is presented in Table. 2 and 3. According to KEGG, rapamycin affects the activity of largest number of targets (84) and aging-associated signaling pathways (10) if compare with other studied substances (Table 2). Wortmannin and PDTC activated 38 and 32 targets, 6 and 4 signaling pathways, respectively. 12 targets and 9 aging associated pathways were activated by 1400W (Table 2). The MAPK and PI3K-Akt signaling pathways were activated by all substances. Caffeine metabolism pathway is activated after exposure to rapamycin, PDTC and 1400W. Signaling pathways Hippo, Hedgehog, Natural killer cell mediated cytotoxicity, and TGF-β are common to rapamycin and wortmannin, whereas Circadian rhythm – for rapamycin and PDTC.

**Table 2:** Genes and aging-associated intracellular signaling pathways, that are activated by the study drugs in Homo sapiens

Drug	Genes*	Longevity pathway IDs**
Rapamycin	GMNN RORC SMAD3 CYP3A4 CYP2D6 CYP2C9 CYP2C19 CYP1A2 DRD2 NR3C1 OPRM1 ESR1 carbonic anhydrase II/CA II PTGS2 ACHE MC4R ESR2 PTSG1 ADRB2 DRD3 SLC6A3 CHRM1 GSK3B NET ABCB1 CHRM3 FLT1 DRD4 CHRM2 CHEK1 ADRB3 ADRB1 CCR5 CCR2 MAOA HRH1 MAPKAPK2 MC3R TBXAS1 MC5R NPY1R CHRM4 ROCK2 PLK1 ACHE AVPR1A NOS2 CHRM5 AKR1B1 MAP2K1 MAP2K1 CHEK2 CYSLTR1 HRH2 NPY2R CCR4 PTPRC MAPKAPK5 CTSG USP1 CYP2A6 ALOX15 ACE MAPKAPK3 MELK PAK4 CALCR VIPR1 LTC4S CSK BCHE SGK1 HIPK2 MYLK HIPK3 SLCO1B1 MAPK15 ROCK2 AKT1 PDCD4	Circadian rhythm Caffeine metabolism Hippo signaling pathway Hedgehog signaling pathway TGF-beta signaling pathway PI3K-Akt signaling pathway MAPK signaling pathway Natural killer cell mediated cytotoxicity mTOR signaling pathway Jak-STAT signaling pathway
Wortmannin	GMNN POLI MAPT NFE2L2 SMAD3 PIN1 VDR IMPA1 GLS RGS4 APOBEC3G WRN GSK3B ERG RECQL CHEK1 MAPKAPK2 ROCK2 PLK1 ACHE MAP2K1 CHEK2 MAPKAPK5 MAPKAPK3 MELK PAK4 CSK BCHE SGK1 HSP90AA1 PLK3 HIPK2 MYLK PLK2 HIPK3 MAPK15 MYLK ROCK2	MAPK signaling pathway TGF-beta signaling pathway Hippo signaling pathway PI3K-Akt signaling pathway Hedgehog signaling pathway Natural killer cell mediated cytotoxicity
PDTC	GMNN ATAD5 POLI MAPT CBX1 NFE2L2 RORC TP53 TXNRD1 CYP3A4 LMNA SMN2 CYP2D6 CYP2C9 VDR CYP2C19 CYP1A2 NPSR1 APOBEC3F RGS4 APOBEC3G carbonic anhydrase II/CA II ALOX15 CHRM1 NFKB1 PMP22 MPHOSPH8 RELA	MAPK signaling pathway Circadian rhythm PI3K-Akt signaling pathway Caffeine metabolism
1400W	FTL CYP3A4 SMN2 CYP2D6 CYP2C9 CYP2C19 CYP1A2 POLK CHRM1 NOS2 NFKB1 NOS2	Caffeine metabolism PI3K-Akt signaling pathway MAPK signaling pathway

**Table 3:** The pharmacological effects of drugs

IDs	Rapamycin	Wortmannin	PDTC	1400W
REACTOME.PATH				
Homo sapiens: Activation of NF-κB in B Cells	4 (1.12e-05)	0 (1)	3 (2.43e-05)	3 (8.73e-06)
Homo sapiens: Cross-presentation of soluble exogenous antigens (endosomes)	3 (0.000144)	0 (1)	3 (9.27e-06)	3 (3.32e-06)
Homo sapiens: Regulation of activated PAK-2p34 by proteasome mediated degradation	3 (0.000144)	0 (1)	3 (9.27e-06)	3 (3.32e-06)
Homo sapiens: Regulation of ornithine decarboxylase (ODC)	3 (0.000153)	0 (1)	3 (9.87e-06)	3 (3.53e-06)
Homo sapiens: Ubiquitin-dependent degradation of Cyclin D	3 (0.000153)	0 (1)	3 (9.87e-06)	3 (3.53e-06)
Homo sapiens: Ubiquitin-dependent degradation of Cyclin D1	3 (0.000153)	0 (1)	3 (9.87e-06)	3 (3.53e-06)
Homo sapiens: CDK-mediated phosphorylation and removal of Cdc6	3 (0.000153)	0 (1)	3 (9.87e-06)	3 (3.53e-06)
Homo sapiens: Vpu mediated degradation of CD4	3 (0.000162)	0 (1)	3 (1.05e-05)	3 (3.76e-06)
Human immunodeficiency virus 1: Vpu mediated degradation of CD4	3 (0.000183)	0 (1)	3 (1.18e-05)	3 (4.23e-06)
Homo sapiens: p53-Independent G1/S DNA damage checkpoint	3 (0.000183)	0 (1)	3 (1.18e-05)	3 (4.23e-06)
Homo sapiens: p53-Independent DNA Damage Response	1 (p > 0.05)	2 (0.00166)	0 (1)	0 (1)
Homo sapiens: Translocation of GLUT4 to the Plasma Membrane	1 (0.0107)	1 (0.00439)	0 (1)	0 (1)
Homo sapiens: Glyoxylate metabolism	1 (0.0128)	1 (0.00527)	0 (1)	0 (1)
Danio rerio: Interleukin receptor SHC signaling	1 (0.017)	1 (0.00702)	0 (1)	0 (1)
Drosophila melanogaster: Purine catabolism	0 (1)	1 (0.00877)	0 (1)	0 (1)
Homo sapiens: GP1b-IX-V activation signalling	1 (0.0212)	1 (0.00877)	0 (1)	0 (1)
Danio rerio: Interleukin-6 signaling	1 (0.0233)	1 (0.00964)	0 (1)	0 (1)
Drosophila melanogaster: Pyrimidine catabolism	1 (p > 0.05)	2 (0.0129)	0 (1)	0 (1)
Homo sapiens: Membrane Trafficking	1 (0.0317)	1 (0.0131)	0 (1)	0 (1)
Danio rerio: Prolactin receptor signaling	0 (1)	1 (0.014)	1 (0.0147)	0 (1)
Mus musculus: N-Glycan antennae elongation	4 (0.000214)	0 (1)	4 (4.89e-06)	3 (8.37e-05)
Homo sapiens: Mitotic G1-G1/S phases	3 (0.000183)	0 (1)	3 (1.18e-05)	3 (4.23e-06)
DOLite				
Skin disease	2 (0.00295)	1 (0.025)	0 (1)	0 (1)
Skin tumor	1 (0.0172)	1 (0.00518)	0 (1)	0 (1)
Histiocytosis	1 (0.022)	0 (1)	0 (1)	0 (1)
Conduct disorder	1 (0.0244)	0 (1)	0 (1)	0 (1)
Dermatitis	2 (0.034)	0 (1)	0 (1)	0 (1)
Infiltrating cancer	1 (0.0341)	0 (1)	0 (1)	0 (1)
Pervasive development disorder	1 (0.0365)	0 (1)	0 (1)	0 (1)
Sarcoma	1 (0.0412)	1 (0.0125)	0 (1)	0 (1)
Skin cancer	1 (0.046)	1 (0.014)	0 (1)	0 (1)
Testicular dysfunction	1 (p > 0.05)	0 (1)	1 (0.0121)	0 (1)
Spondylarthropathies	0 (1)	0 (1)	0 (1)	1 (0.00222)
Arthritis	0 (1)	0 (1)	0 (1)	1 (0.016)
GO.BP				
Regulation of cellular amino acid metabolic process	3 (0.000158)	0 (1)	3 (3.19e-06)	3 (2e-06)
DNA damage response, signal transduction by p53 class mediator resulting in cell cycle arrest	3 (0.000232)	0 (1)	3 (4.72e-06)	3 (2.96e-06)
Signal transduction involved in mitotic cell cycle checkpoint	3 (0.000232)	0 (1)	3 (4.72e-06)	3 (2.96e-06)
Signal transduction involved in mitotic cell cycle G1/S transition DNA damage checkpoint	3 (0.000232)	0 (1)	3 (4.72e-06)	3 (2.96e-06)
Signal transduction involved in mitotic cell cycle G1/S checkpoint	3 (0.000232)	0 (1)	3 (4.72e-06)	3 (2.96e-06)
Negative regulation of ubiquitin-protein ligase activity involved in mitotic cell cycle	3 (0.000242)	0 (1)	3 (4.94e-06)	3 (3.1e-06)
Signal transduction involved in DNA integrity checkpoint	3 (0.000242)	0 (1)	3 (4.94e-06)	3 (3.1e-06)
Signal transduction involved in G1/S transition checkpoint	3 (0.000242)	0 (1)	3 (4.94e-06)	3 (3.1e-06)
Signal transduction involved in DNA damage checkpoint	3 (0.000242)	0 (1)	3 (4.94e-06)	3 (3.1e-06)
Regulation of cellular ketone metabolic process	4 (0.000249)	0 (1)	3 (8.22e-05)	3 (5.18e-05)
Establishment of protein localization to organelle	2 (p > 0.05)	3 (2.87e-05)	0 (1)	0 (1)
Cellular protein localization	3 (p > 0.05)	4 (8.85e-05)	0 (1)	0 (1)
Cellular macromolecule localization	3 (p > 0.05)	4 (9e-05)	0 (1)	0 (1)
Protein targeting to mitochondrion	1 (p > 0.05)	2 (0.000131)	0 (1)	0 (1)
Establishment of protein localization to mitochondrion	1 (p > 0.05)	2 (0.000156)	0 (1)	0 (1)
Protein localization to mitochondrion	1 (p > 0.05)	2 (0.000162)	0 (1)	0 (1)
Protein targeting	2 (p > 0.05)	3 (0.000334)	0 (1)	0 (1)
Oxalic acid secretion	1 (0.00183)	1 (0.000339)	0 (1)	0 (1)
Protein localization to organelle	2 (p > 0.05)	3 (0.000404)	0 (1)	0 (1)
Protein localization	3 (p > 0.05)	4 (0.000642)	1 (p > 0.05)	0 (1)
Signal transduction involved in cell cycle checkpoint	3 (0.000253)	0 (1)	3 (5.17e-06)	3 (3.24e-06)
Number of genes	75	35	26	11

*Legend*: «↑» - increasing, «↓» - decreasing, “=” – no effect, “-” - not studied, M - median lifespanl, 90% - age of 90% mortality; LA - locomotor activity; F - fertility; *p <0.05, **p <0.01, Gehan–Breslow–Wilcoxon test and Mantel–Cox test for M and Wang-Allison test for 90%.

According to REACTOME.PATH the strongest pharmacological activity was revealed for rapamycin, PDTC and W1400. It should be noted the effect of rapamycin, PDTC and 1400W on the activity of proteasome, NF-κB and ornithine decarboxylase, as well as on the regulation of endocytosis, cell cycle and mitosis in humans (Table 3). Treatment of rapamycin and wortmannin affect the glyoxylate metabolism and cellular uptake of glucose in humans (Table 3). In *Drosophila* wortmannin affect the metabolism of DNA and RNA (the catabolism of purines and pyrimidines) (Table 3). DOLite identified associations between wortmannin, rapamycin and different tumor types, which characterizes them as antitumor drugs (Table 3). GO.BP shows the effect of rapamycin, PDTC and 1400W on cell metabolism, DNA damage response and cell cycle control (Table 3).

## DISCUSSION

### Effects on the lifespan and life quality

According to our analysis of the literature is being discovered more than 100 pharmaceutical substances that can prolong the lifespan of model organisms. However, the increase of lifespan with aging-suppressor substances rarely exceeds 40% [50, 51], which greatly less than effects (up to 1000% or more) caused by mutations in the regulatory genes, which are the key switches of cell program to maintain growth or resist to stress, such as gene of PI3Ksubunit [52]. We proceeded on the assumption that a more effective aging-suppressor drugs may be substances with specificity to the products of genes that control the evolutionarily conserved mechanisms of aging, mutations in which have the greatest effect on lifespan and the aging rate. In this regard, we investigated the aging-suppressive properties of specific pharmacological inhibitors of aging associated gene products TOR, PI3K, NF-κB and iNOS. To date, in *Drosophila melanogaster* described homologs of TOR [53], PI3K [54] and NF-κB orthologs Relish [55] and Dorsal [56]. *Drosophila* NO synthase gene dNOS encodes a protein that bears a strong resemblance to all three NOS isoforms of mammalshas [57]. The presence of target proteins in *Drosophila* make possible to use it as a model for pharmacological screening of substances with the proposed aging-suppressor activity.

In this study, we revealed the aging-suppressive effect of rapamycin in nanomolar concentrations (0.005 μM), in which it significantly increases the median lifespan of females (by 14%) and females (by 12%) and improves quality of life in test on locomotor activity. Previously it was shown that pharmacological inhibition of TOR prolongs lifespan in yeast [58], fruit flies [11, 12] and mice [16, 18]. It has been shown the aging-suppressor activity of rapamycin in concentrations of 10, 20 and 40 nM in yeast [58]. In *Drosophila* the lifespan increasing effect of rapamycin was accompanied by resistance to oxidative stress and starvation [11], and mice observed increase in locomotor activity aging males [18]. However, in *Drosophila* the lifespan increasing effect of rapamycin in concentrations of 50, 200 and 400 μM was accompanied by a significant reduction in fertility [11]. This data demonstrate that lifespan increasing effect of rapamycin in micromolar concentrations may be due to redistribution of resources from reproduction to longevity and stress resistance. We found out that in nanomolar concentrations rapamycin increase lifespan without negative effect on fertility.

Specific inhibition of PI3K by wortmannin in concentration of 0.005 μM do not lead to a statistically significant effect on lifespan. Wortmannin at a concentration of 5 μM increased the median lifespan of males (by 5%), but decreaseв it in females (by 8.2%). At the same time wortmannin in concentrations of 0.005 μM and resulted in increased locomotor activity in males. The wortmannin toxicity that was observed in females in a relatively high concentration 5 μM may be related to its irreversible binding of the drug to essential proteins other than PI 3-kinase (wortmannin is known to bind irreversibly to its target [59]).

We have previously shown that wortmannin (0.5 μM) as well as another PI3K inhibitor LY294002 (5 μM) increased the median and maximal lifespan in *Drosophila* males and females [12, 21]. Additionally, treatment with wortmannin (5 μM) and LY294002 (5, 100 μM) resulted in an increase in survival of *Drosophila* male and female after acute exposure to ionizing radiation in a dose of 30 Gy [60]. Thus, the lifespan effect of the PI3K inhibitors is concentration dependent and is apparently determined by involvement of this enzyme in maintaining of delicate balance between the development/reproduction on the one hand and stress/longevity on the other. In addition to the concentration dependence we observed the differences of PI3K inhibition effects in different sexes.

According to literature data, specific inhibition of PI3K by LY294002 leads to increasing of lifespan in rotifers [61], induces dauer formation, thermotolerance and longevity in nematodes [20]. *Caenorhabditis elegans* strains bearing homozygous nonsense mutations in the *age-1* gene, which encodes the catalytic subunit of PI3K (PI3K_CS_), produce extremely long-lived progeny with nearly 10-fold extension of both median and maximum adult lifespan relative to N2DRM, a long-lived wild-type stock into which the null mutant was outcrossed [52]. At the same time PI3K-null worms have prolonged developmental times, increased resistance to oxidative and electrophilic stresses [52]. The ablation of insulin-like peptide-producing median neurosecretory cells in *Drosophila* brain leads to reduced fecundity, and reduced tolerance of heat and cold [62]. However, ablated flies show an extension of median and maximal lifespan and increased resistance to oxidative stress and starvation [62].

In all variants of our experiments treatment by NF-κB inhibitor PDTC (1.25, 12.5 and 125 μM) increased the median lifespan of males (by 6-10%). PDTC in concentrations of 1.25 and 12.5 μM decreased median lifespan of females by 2%, but in 125 μM increased by 12%. Treatment with QNZ (0.03, 0.3, 3 μM) did not affect the lifespan of males. In females, treatment with 0.03, 0.3 and 3 μM of QNZ reduced median lifespan by 4-15%. The differences in the effects of PDTC and QNZ may be associated with different molecular mechanisms of action of these compounds. While PDTC inhibits NF-κB activation by preventing degradation I-κB [63, 64], QNZ inhibits the NF-κB at the transcriptional activity level [65]. Furthermore, PDTC has antioxidant and metal chelating properties [63, 64], which may enhance its aging-suppressive action.

Recent studies suggest that the NF-κB transcription factor controls age-dependent changes in inflammation genes expression. For example the increase of NF-κB dependent genes expression in human blood vessel endothelium with age is primarily linked to decreased IκB-mediated NF-κB inhibition [66]. With age expression of NF-κB dependent genes contributing to progression of atherosclerosis in rat glomeruli increases [67]. However, selective inhibition of NF-κB activity in blood vessel endothelial cells prevents atherosclerosis progression [68]. Genetic blockade of NF-κB in the skin of chronologically aged mice reverses the global gene expression program and tissue characteristics to those of young mice [69, 70]. Chronic NF-κB mediated immune system activation enhanced pathogen resistance, however significantly reduced lifespan [71]. Extensive evidence has now emerged indicating a critical role for NF-κB in promoting oncogenic conversion and in facilitating later stage tumor properties such as metastasis [34]. Pharmacological inhibitors of NF-κB pathways often, but not always, suppress cancer growth [34]. Decrease of NF-κB activity level impedes progression of degenerative phenotype in mice with knocked out *Sirt6* sirtuin gene that is involved in the base excision repair [72]. It is suggested that the most important homeostatic function of SIRT6 is in the prevention of NF-κB dependent gene overactivation via deacetylation of lysine 9 of the H3 histone (H3K9) on the promoters of NF-κB target genes, whereas overactivation of NF-κB promotes normal and accelerated aging [72]. Pharmacological inhibition of IκB kinase (IKK) leads to a delay of the development of age-related pathologies in mice with progeria, reduces the level of oxidative DNA damage and prevents stress-induced cellular senescence [73]. Recently it was revealed that IKK-β and NF-κB inhibit gonadotropin-releasing hormone (GnRH) to mediate ageing-related hypothalamic GnRH decline [27]. After age-dependent inhibition of IKK-β activation and NF-κB in the hypothalamus or in the brain of mice delay the aging process and increase the lifespan (by 20%) [27]. Thus IKK-β and NF-κB mediate the programmatic role of hypothalamus in ageing development via immune-neuroendocrine integration [27]. Overactivation of innate immune-response pathways in the brain is responsible for neurodegeneration [74]. In *Drosophila* neurodegeneration is dependent on the NF-κB transcription factor, Relish [74]. In our earlier studies it has been shown the lifespan increasing in *Drosophila* when exposed to PDTC in a concentration of 125 μM [22].

The observed increase in lifespan in males and its decrease in females inhibiting iNOS with (0.03, 0.3, 3 μM) may be due to the ambiguous role of this enzyme in aging and longevity. For example, iNOS gene knockout mice have reduced lifespan, but the increased activity of this gene leads to increased risk of cardiovascular diseases [75]. Pharmacological inhibition of iNOS leads to reduced risk of cardiovascular disease in rats [76]. Consequently, as overactivation well as complete suppression of iNOS lead to negative effects. Since females consume more food than males, they get more substance during lifetime [77]. This can lead to excessive inactivation of dNOS and negative effects on lifespan and locomotor activity. The lifespan decreasing in females may be caused by an up-regulation of reproduction. Thus, it can be assumed that the lifespan effect of 1400W in different concentration was gender specific and associated with the amount of consumed substance and rate of reproduction.

Nitrosative stress is now considered as an important cause of physiological decline that characterizes the aging of many tissues [29, 78]. Age related changes in nitric oxide synthase activity and/or expression and the consequent changes in NO production and biological activity, apparently contribute to this reduction [29, 78]. Several recent studies suggest that NO and iNOS could be involved in aging-induced insulin resistance [79] as well as in cardiovascular, pulmonary, musculoskeletal neurological dysfunction, and cancer [78, 80]. It has been shown that iNOS activation can inhibit DNA repair pathways including direct DNA repair, base and nucleotide excision repair [80]. As it is well known DNA repair pathways play the crucial role in aging process and development of age-related pathologies [81]. Thus, genetic, pharmacological, and physiological iNOS inhibition may contribute to the combat against age-related diseases and lifespan increasing.

Since the aging is a complex process that involves many intracellular signaling pathways, we made the assumption that the most pronounced effect on longevity may be achieved by simultaneous inhibition of several aging-associated signaling pathways. According to our data, the combined effects on longevity of rapamycin (5 μM) and wortmannin (5 μM) and the combination of PDTC (125 μM) with rapamycin (0.005 μM) and wortmannin (0.005 μM) is female-biased. In males, the combined application of substances increase lifespan no greater than each of the inhibitors tested separately. The gender difference of the observed effects may be due to the initially existing sex differences in drug metabolisms [25], stress resistanse [82] reproductive strategies [83]. It is shown that combined treatment of nematodes by curcumin and thioflavin T did not demonstrate additive effect on the lifespan [84].

### Bioinformatic analysis

Analysis of targets that are affected by drugs according KEGG shows, that compounds affect MAPK and PI3K-Akt signaling pathway (all compounds), Caffeine metabolism (rapamycin, PDTC and 1400W) and Circadian rhythm pathways (rapamycin and PDTC) that are involved in lifespan control [5, 85, 86] and cancer development [87-89]. Rapamycin and wortmannin affected Hippo and Hedgehog that are tumor suppression and longevity associated pathways [90, 91].

REACTOME.PATH analysis showed that rapamycin, PDTC and 1400W influence such aging-related processes such as activation of NF-κB, regulation of endocytosis, cell cycle and mitosis. Furthermore rapamycin, PDTC and 1400W affect regulation of ornithine decarboxylase, associated with aging and Alzheimer's disease [92], lymphoma [93] and breast cancer [94]. Rapamycin and wortmannin affect the regulation of the cellular uptake of glucose and glyoxylate metabolism. Expression of the main glyoxylate enzyme is up-regulated in *daf-2* and *age-1* long-lived *Caenorhabditis elegans* mutants [95, 96]. Wortmannin affects the catabolism of purines and pyrimidines involved in the processes of aging through DNA and RNA metabolism, maintaining energy balance [97].

Analysis of gene-associated diseases by DOLite also reveals an association of rapamycin and wortmannin with genes involved in the molecular mechanisms of different types of cancer. Wortmannin is associated with skin tumor, while rapamycin with skin tumor, histiocytosis, infiltrating cancer, sarcoma. These data confirm the anti-cancer properties of these aging-suppressor drugs and a close relationship between aging and carcinogenesis.

According to GO.BP analysis rapamycin activates the DNA damage response that triggers p53-dependent cell cycle arrest. While arresting cell cycle, p53 may simultaneously suppress the senescence program, thus causing quiescence. p53 suppresses senescence presumably by inhibition of mTOR [98, 99]. Senescence occurs when p53 fails to inhibit mTOR. In addition it was shown that rapamycin extended the mean lifespan of p53+/− mice and decreased the incidence of spontaneous tumors [17]. Thus, rapamycin may compensate decreasing of p53 function, that prevents senescence, extends lifespan and decreases the rate of tumors via TOR-dependent mechanism. Recently, we found that overexpression of the *GADD45* gene leads to an increase in longevity and stress resistance of *Drosophila* [100, 101]. *GADD45* A in mammals is a p53-activated gene [102]. In this context the p53-dependent activation of *GADD45* may complete the anti-aging and stress resistance effects of p53.

The bioinformatic analysis of data obtained from KEGG, REACTOME.PATH, DOLite and GO.BP revealed the highest activity of rapamycincin in comparison with wortmannin, PDTC and 1400W.

The promising strategy to slow down ageing and prevent or delay the onset of age-related diseases is that of mild stress-induced hormesis by using hormetins [103]. Previously we observed lifespan expanding effect of radiation hormesis [104], that may be mediated by *FOXO*, *SIRT1*, *JNK*, *ATM*, *ATR*, and *p53* genes [105] as well as heat shock proteins and heat shock factor [106]. The results of bioinformatics analyses demonstrate that rapamycin, wortmannin, PDTC, QNZ and 1400W induce aging suppressive effects as hormetins that inhibit the aging-associated pathways [107]. Obviously that studied drugs inhibit the hyperactivity of pro-aging signaling. Thus our data fully agree with hyperfunction theory of Mikhail Blagosklonny [26, 36].

Thus, we conducted a comprehensive study of rapamycin, wortmannin, PDTC, QNZ and 1400W effects in different concentrations and combinations on the life span and quality of *Drosophila*. Our data demonstrates that inhibiton of PI3K, TOR, NF-κB and iNOS may increase life span without decreasing of the life quality (fertility and locomotor activity). The low concentrations of inhibitors are less likely induce negative effects on the quality of life. Also, we found significant differences in the effects of inhibitors treatment between males and females. In females, we observed a more negative effects on longevity without reducing the locomotor activity or fertility. In all experimental variants treatment of males increased life span and locomotor activity was not lower than in the control group. It has been found that the combined effect of drugs has the largest positive effect on the life span of females. The differences observed between males and females may be due to the functional features of the sexes at the level of the genome, transcriptome and metabolome.

## MATERIALS AND METHODS

### Drosophila strains

We used wild-type *Canton-S* flies for the experimental procedures (provided by the Bloomington *Drosophila* Stock Center at Indiana University, Bloomington, Indiana, USA).

### Treatment with inhibitors

We greased fly medium by paste of hydrolyzed yeast containing one of the substances. Control untreated animals were fed by yeast past without substances. To make the hydrolyzat yeast were boiled in water bath for 30 minutes. To prepare the 100 ml of paste 50 g of dry yeast per 60 mL of water were used.

The following compounds were fed to imago flies through yeast paste in a throughout the lifetime:

- rapamycin (Sigma-Aldrich, USA) (Rapa) – TOR inhibitor [108], 0.005 μM;

- wortmannin (Sigma-Aldrich, USA) (Wm) – PI3K inhibitor [109], 0.005 and 5 μM;

- pyrrolidine dithiocarbamate (Sigma-Aldrich, USA) (PDTC) – NF-κB inhibitor [63, 64], 125 μM; 12.5 μM; 1.25 μM;

-N4-[2-(4-phenoxyphenyl)ethyl]-4,6-quinazolinediamine (Merck KGaA, Germany) (QNZ) – ингибитоp NF-κB inhibitor [65], 3 μM, 0.3 μM, 0.03 μM;

- N-(3-(Aminomethyl)benzyl)acetamidine (Merck KGaA, Germany) (1400W) – iNOS inhibitor [110], 3 μM, 0.3 μM, 0.03 μM;

- mix 5 μM Rapa + 5 μM Wm;

- mix 125μM PDTC + 0.005 μM Rapa;

- mix 125μM PDTC + 0.005 μM Wm.

### Lifespan analysis

Control and experimental flies were maintained at 25±0.5°C in a 12 h-12 h light/dark cycle on a sugar-yeast medium covered with the yeast paste. To estimate the longevity 250-450 flies were collected during 24 h since the onset of eclosion (about 30 adult flies per 120 ml vial) for each experimental variant. Males and non-virgin females were kept separately. Flies were transferred to a fresh medium two times a week. Dead flies were counted daily. For each experimental variant 2 biological replicates were pooled.

Survival functions were estimated using the Kaplan–Meier procedure and plotted as survival curves [111]. Median lifespan and the age of 90% mortality were calculated. The statistical analysis of survival data was conducted using nonparametric methods. Comparison of survival functions was done using the modified Kolmogorov–Smirnov test [112]. The statistical significance of differences between the mean life spans for the experimental and control variants was determined using the Gehan–Breslow–Wilcoxon [113] and Mantel–Cox tests [114]. To test the statistical significance of differences in maximum lifespan (age of 90% mortality), the Wang–Allison test was used [115]. The Kaplan-Meier curves were plotted using STATISTICA, version 6.1 (StatSoft Inc, USA). Calculation of lifespan parameters and their statistical analysis were performed in the R software environment for statistical computing and graphics (http://www.r-project.org/).

### Fertility assay

Fertility by number of laid eggs and number of pupae was estimated. The flies were kept under the same conditions (10 females and 10 males per vial) and transferred to a fresh medium twice a week. Daily egg production by females was calculated once a week. After 10 days, formed pupae were counted. The significance of differences between fertility of control and treated flies at different ages was evaluated using the *x*^2^-test.

### Locomotor activity assay

To estimate the locomotor activity parameters, 90 flies were collected (30 flies per vial) for each experiment. Flies were kept under identical conditions and transferred to a fresh medium twice a week. Males and females were studied separately. Measurements of locomotor activity were carried out using the *Drosophila* Population Monitor (TriKinetics Inc, USA). For the evaluation of spontaneous activity the total activity of the 30 flies for 3 minutes was taken into account. To test negative geotaksis flies were flicked on the bottom of the vial and the movement was measured during 20 seconds. The arithmetic mean of three replicates was considered. The significance of differences between locomotor activity (a total number of infrared beam crossings) in sample groups at different ages was evaluated using the *x*^2^-test [116].

### Bioinformatic analysis

Also, we carried out the bioinformatic analysis of rapamycin, wortmannin, PDTC and 1400W effects. Identification of drug targets were performed using the ChEMBL resource (https://www.ebi.ac.uk/chembldb/compound) [117]. To analyze the functional characteristics of the targets a comparisons of the KEGG were performed. KEGG - is a method of molecular pathways annotations where particular gene is involved in, that provided by the Kyoto Encyclopedia of Genes and Genome (www.genome.jp/kegg) [118]. Also the information from REACTOME.PATH, DOLite и GO.BP databases were used. The basic unit of the REACTOME.PATH database is reaction. The reactions are grouped in the causal chain in the form of a path (http://www.reactome.org) [119]. DOLite - is a database of gene-disease associations, which allows to track the relationship between genes and pathologies (http://fundo.nubic.northwestern.edu/) [120]. GO - is a project to unify the representation of gene and gene product attributes across all species (http://www.geneontology.org/) [121]. The objectives of the GO project are to compile annotations to the genes and products, maintaining and updating a clearly defined list of gene attributes and their products in the domains «biological process», «molecular function», «cellular component». Obtaining of gene ontologies for lists of considered genes was performed in the R package BioMart [122, 123]. Analysis and comparison of the investigated substances were performed in the R package GeneAnswers for annotation of the molecular mechanisms proposed by REACTOME.PATH, DOLite and GO.BP [124].

## Supplementary Figures


