# Supplementary material for: Selective anticancer agents suppress aging in Drosophila
Source: Oncotarget. 2013 Sep 7;4(9):1507–26. doi: 10.18632/oncotarget.1272 (PMC3824538; doi:10.18632/oncotarget.1272)
Supplement: Supplementary file 1 [file oncotarget-04-1507-s001.pdf]

Selective anticancer agents suppress aging in *Drosophila* - Danilov et al

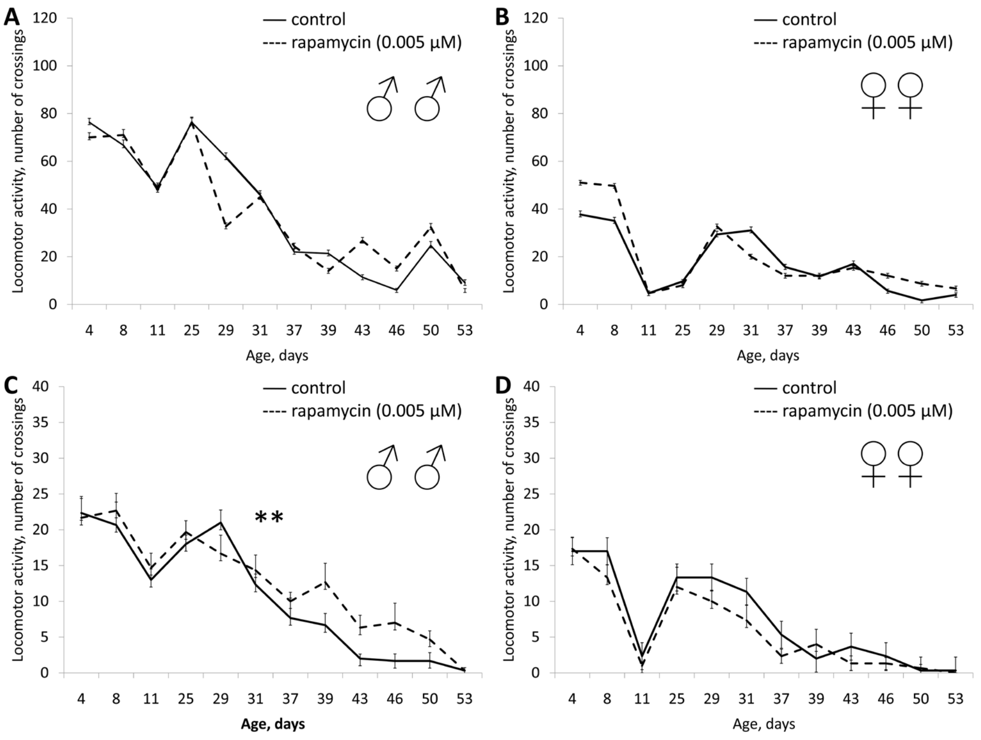

Figure S1: Effect of rapamycin (0.005 µM) on locomotor activity Drosophila melanogaster. (A, B) spontaneous activity. (C, D) negative geotaksis test. * p< 0.001, ** p< 0.05 (χ^2 test).

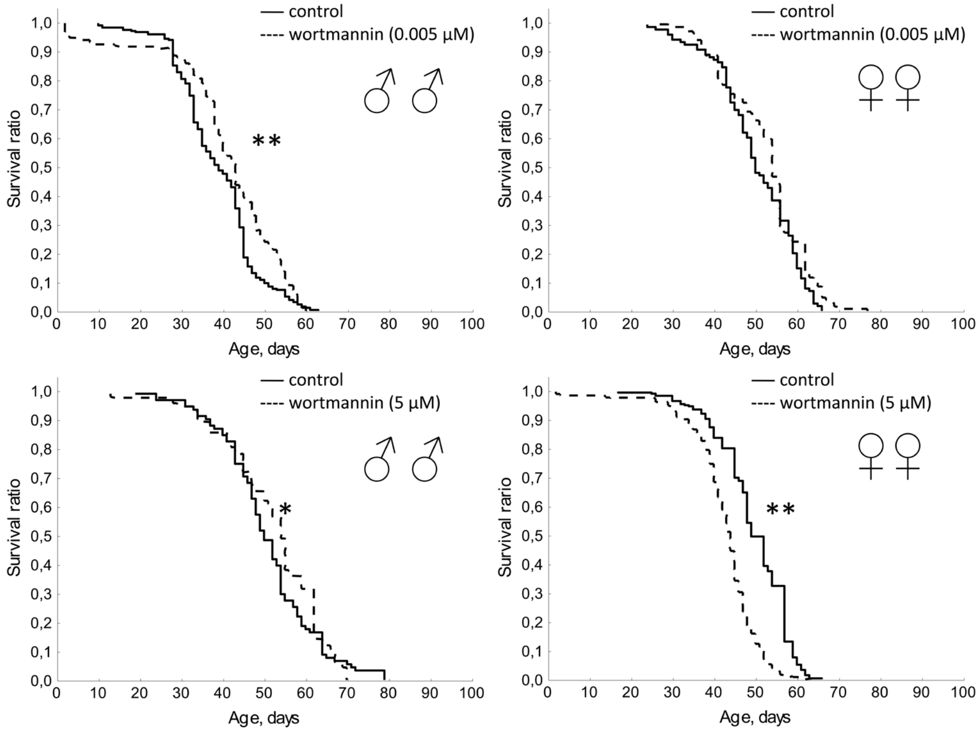

Figure S2: Effect of wortmannin (0.005 and 5 µM) on lifespan Drosophila melanogaster. * p< 0.001, ** p< 0.05 (Kolmogorov-Smirnov test).

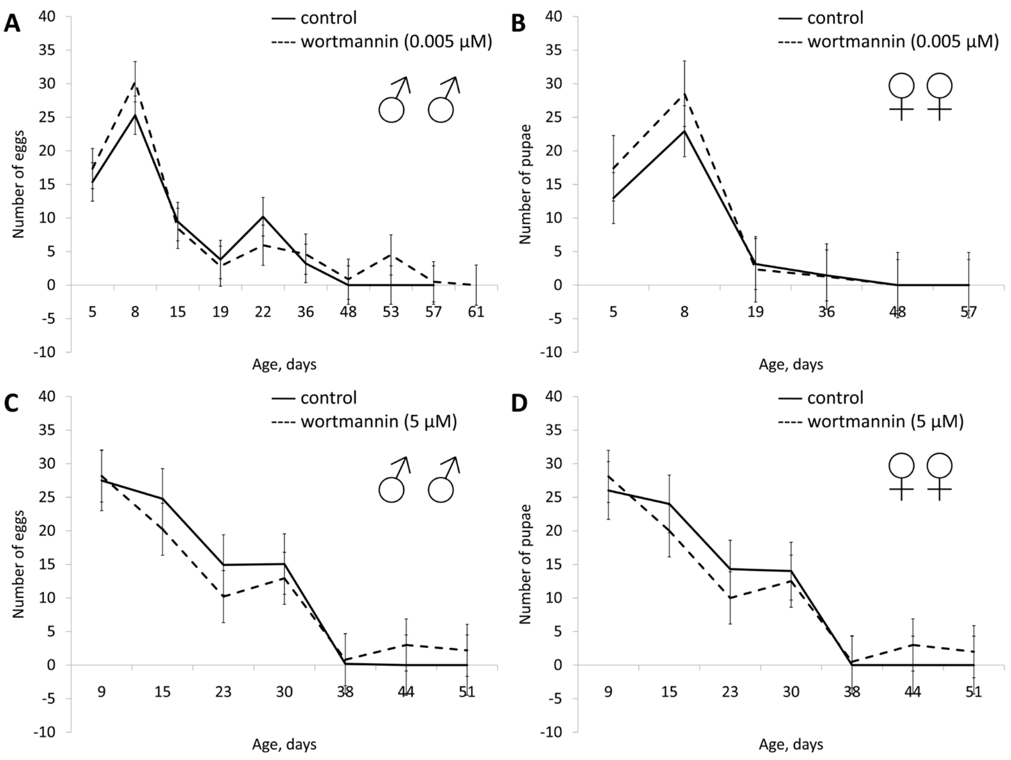

Figure S3: Effect of wortmannin (0.005 and 5 μM) on fertility of females Drosophila melanogaster. * $p< 0.001$, ** $p< 0.05$ (χ^2 test).

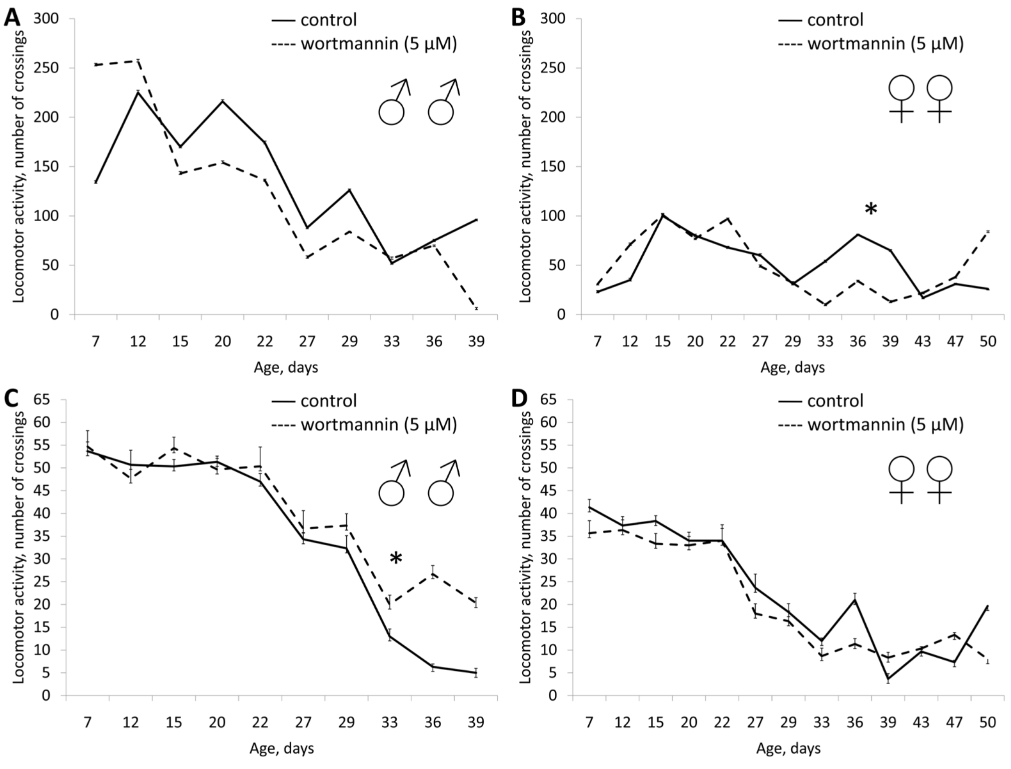

Figure S4: Effect of wortmannin (5 μM) on locomotor activity Drosophila melanogaster. (A, B) spontaneous activity. (C, D) negative geotaksis test. * $p< 0.001$, ** $p< 0.05$ (χ^2 test).

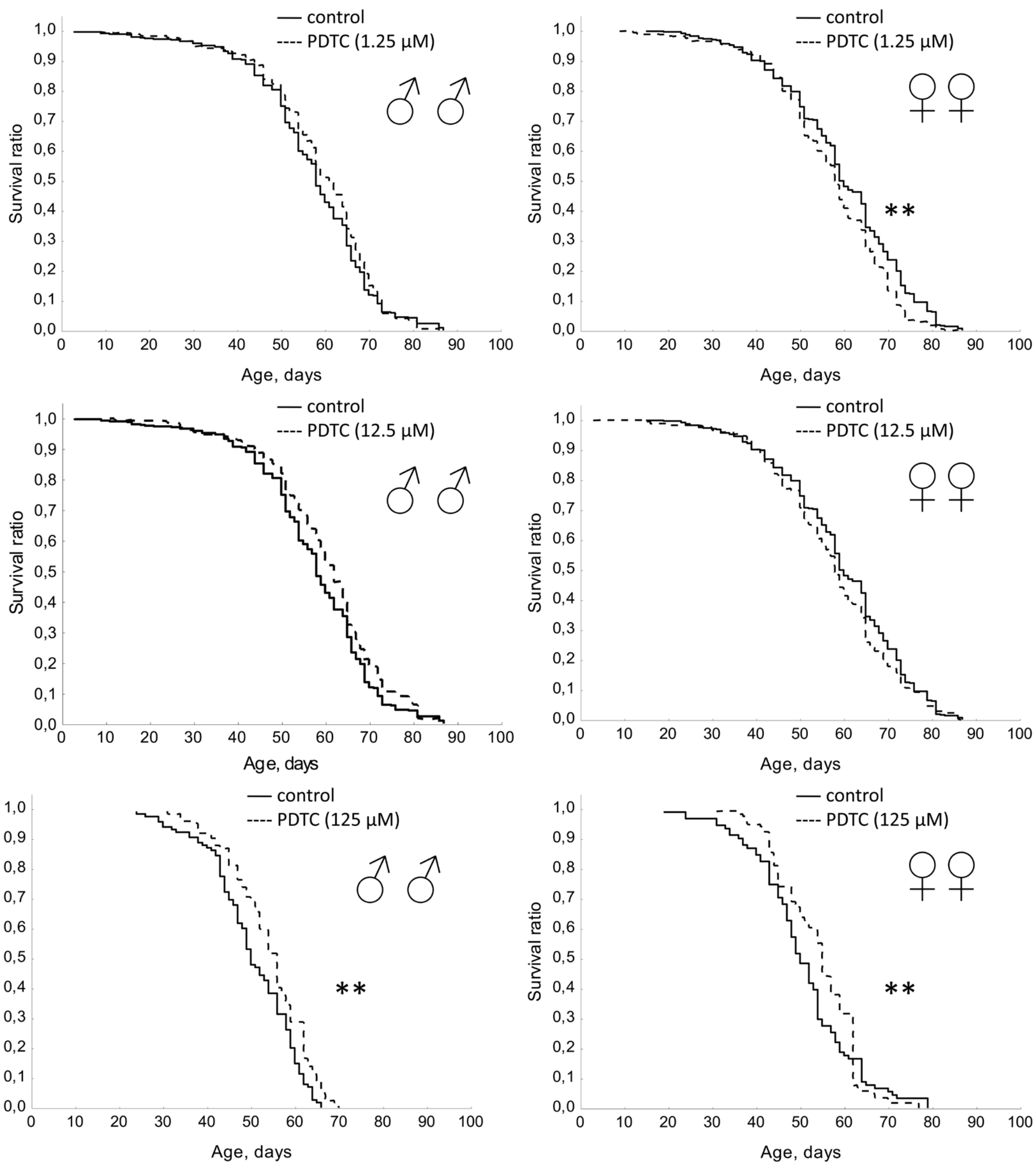

Figure S5: Effect of PDTC (1.25, 12.5, 125 µM) on lifespan Drosophila melanogaster. * p< 0.001, ** p< 0.05 (Kolmogorov-Smirnov test).

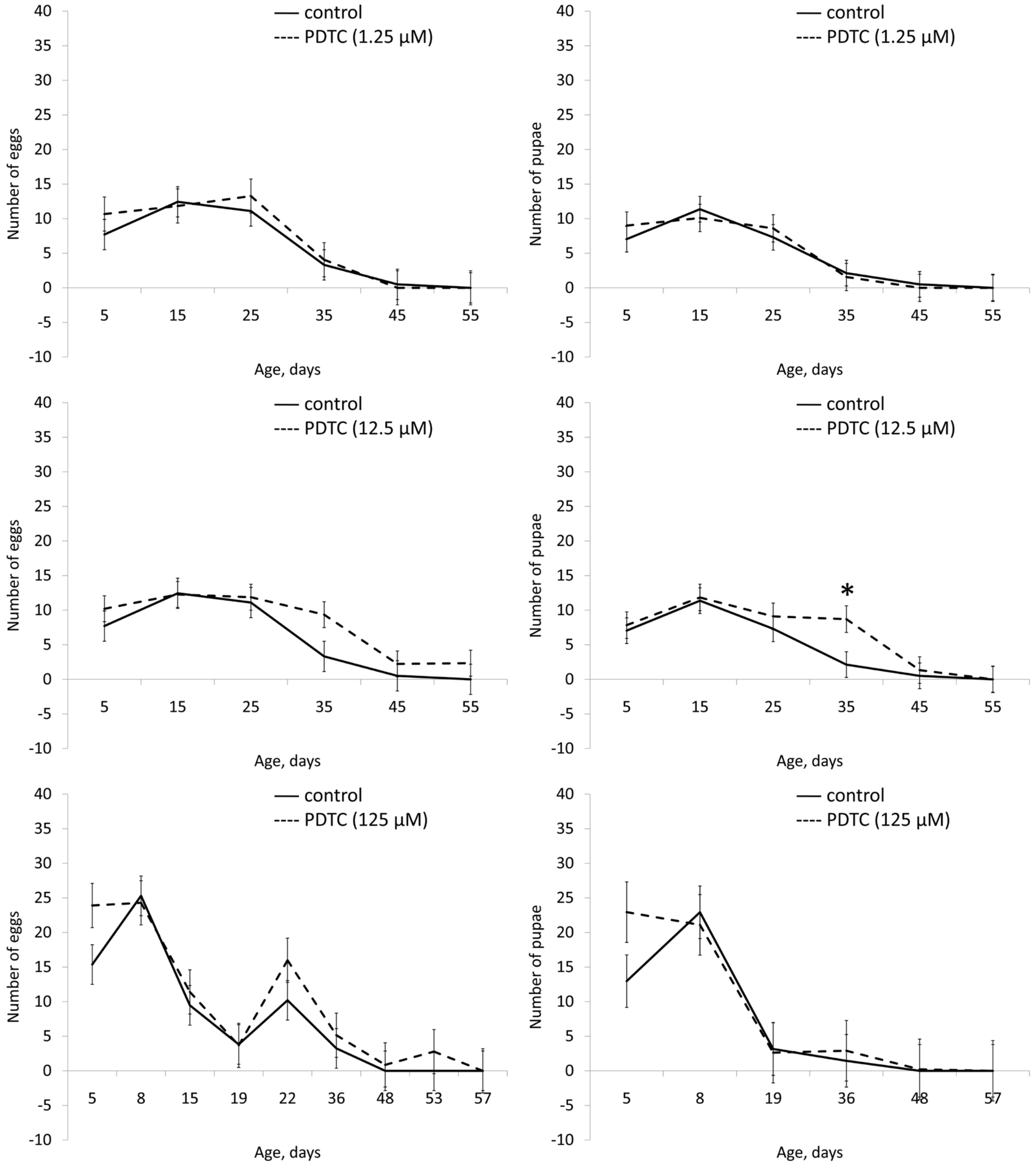

Figure S6: Effect of PDTC (1.25, 12.5, 125 µM) on fertility of females Drosophila melanogaster. * p< 0.001, ** p< 0.05 (χ^2 test).

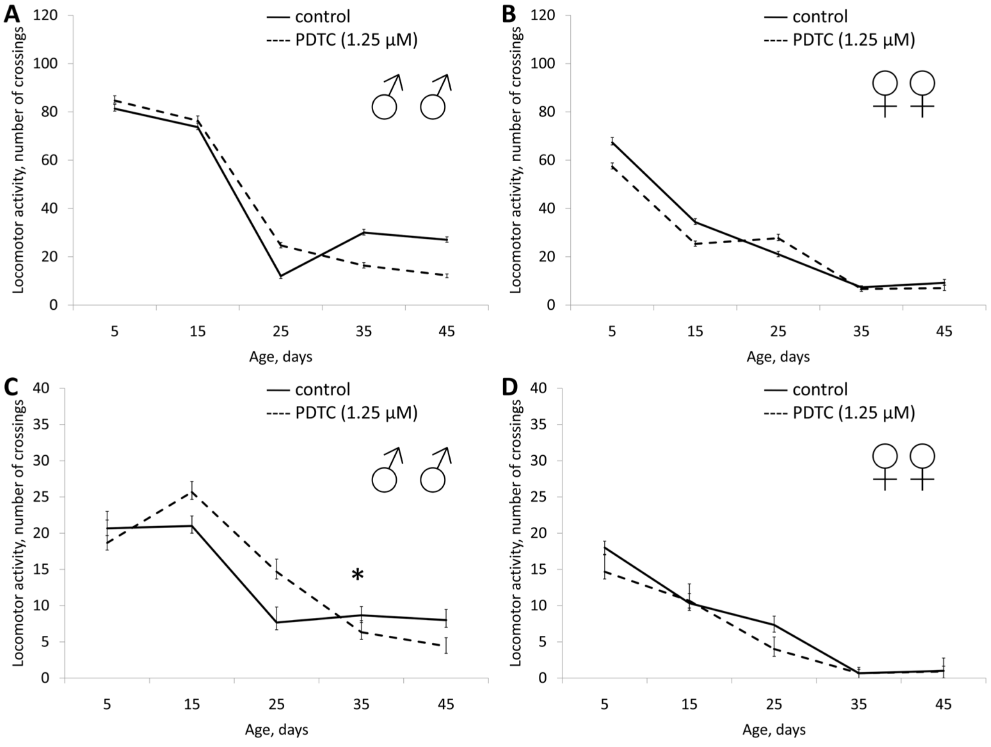

Figure S7: Effect of PDTC (1.25 µM) on locomotor activity Drosophila melanogaster. (A, B) spontaneous activity. (C, D) negative geotaksis test. * p< 0.001, ** p< 0.05 (χ^2 test).

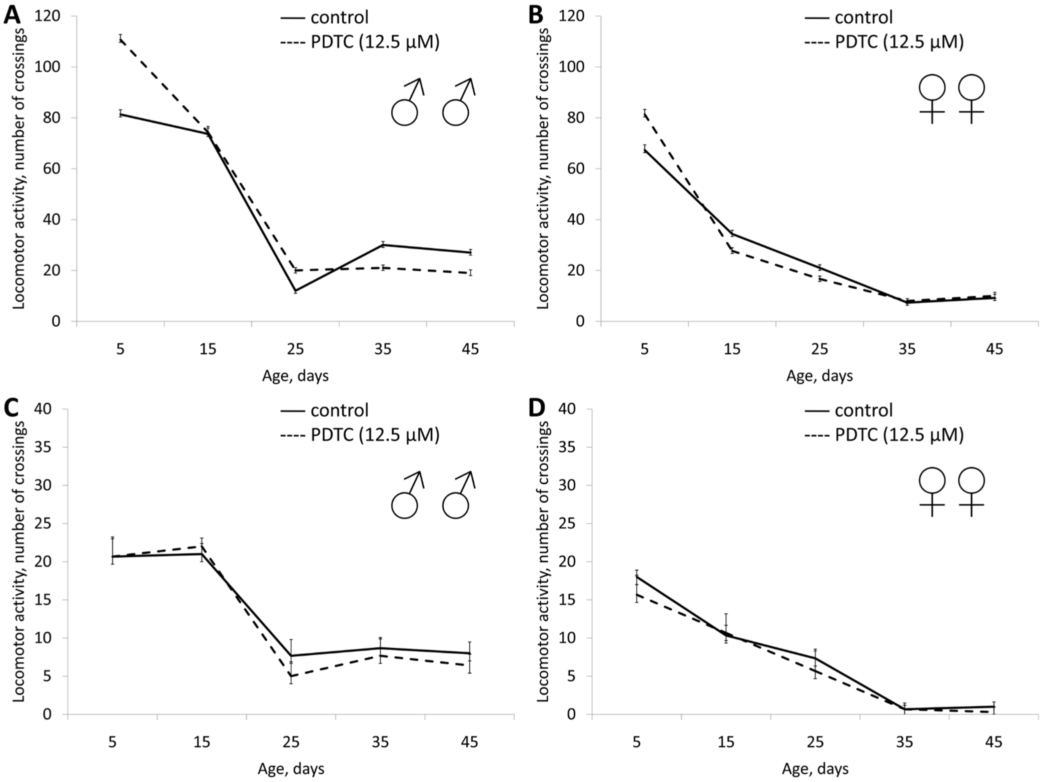

Figure S8: Effect of PDTC (12.5 µM) on locomotor activity Drosophila melanogaster. (A, B) spontaneous activity. (C, D) negative geotaksis test. * p< 0.001, ** p< 0.05 (χ^2 test).

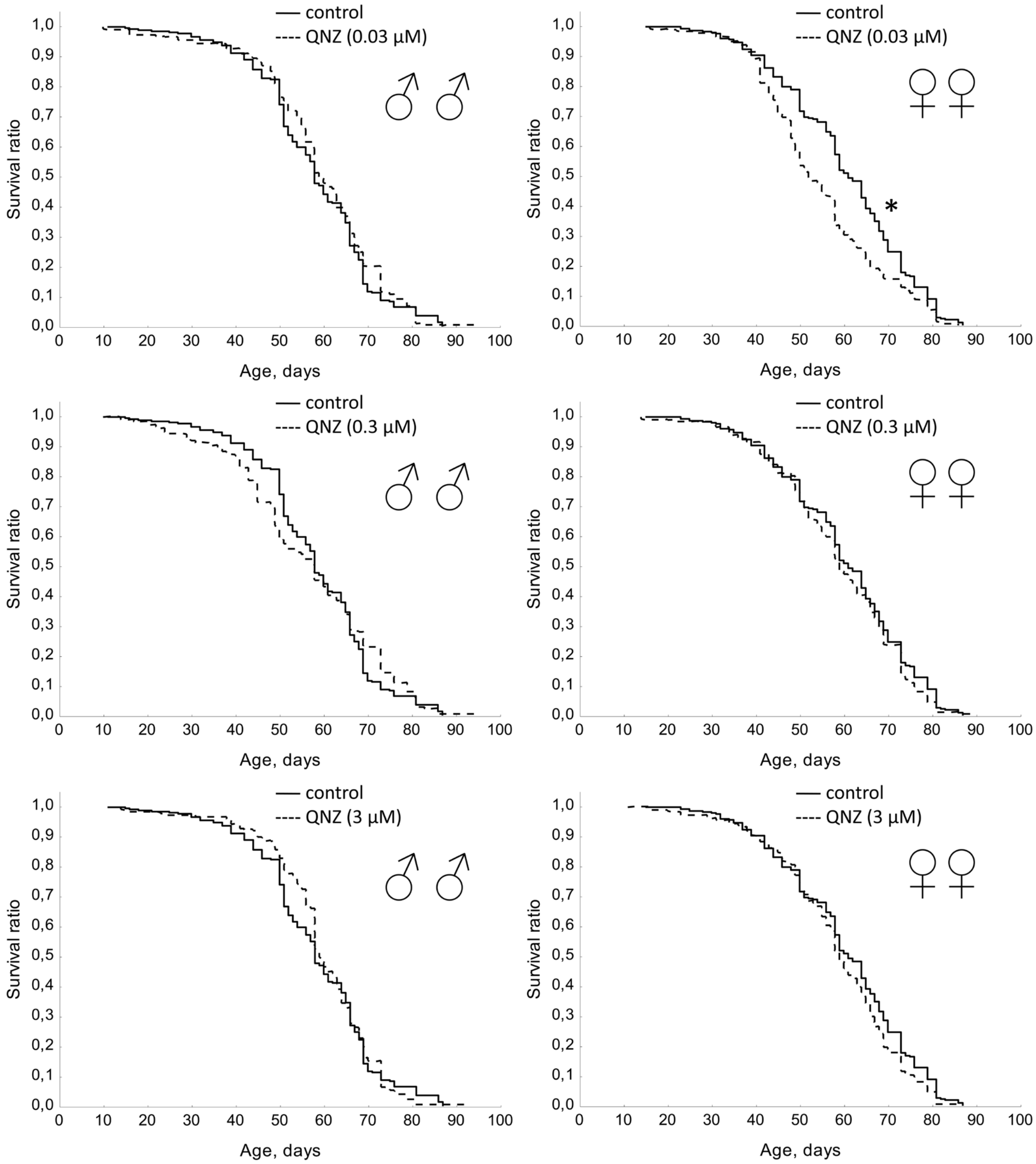

Figure S9: Effect of QNZ (0.03, 0.3, 3 µM) on lifespan Drosophila melanogaster. * p< 0.001, ** p< 0.05 (Kolmogorov-Smirnov test).

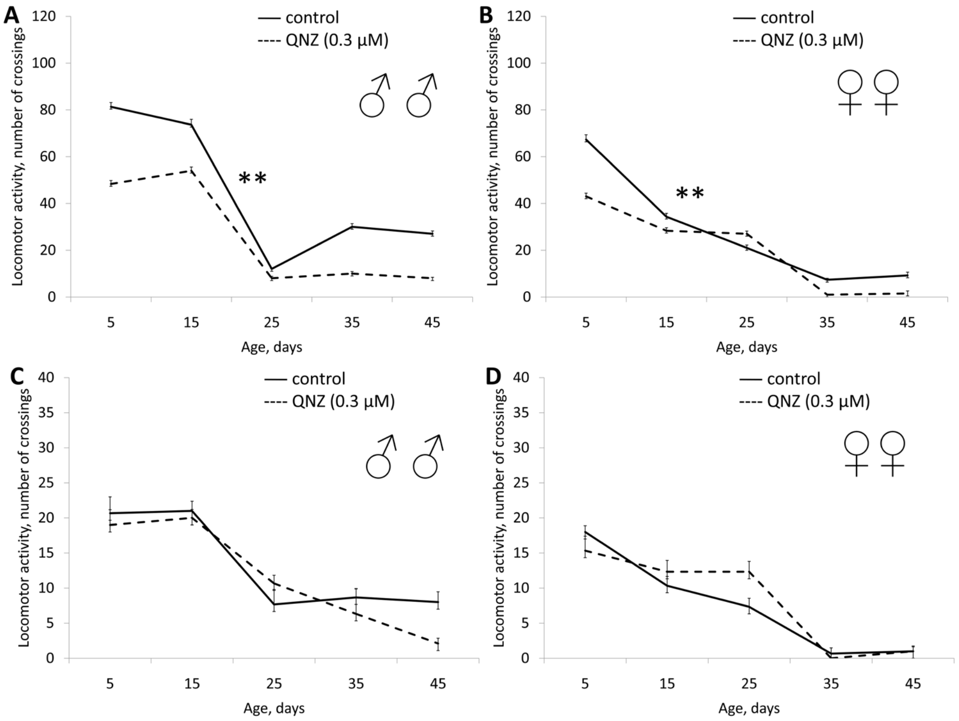

Figure S10: Effect of QNZ (0.3 µM) on locomotor activity Drosophila melanogaster. (A, B) spontaneous activity. (C, D) negative geotaksis test. * p< 0.001, ** p< 0.05 (χ^2 test).

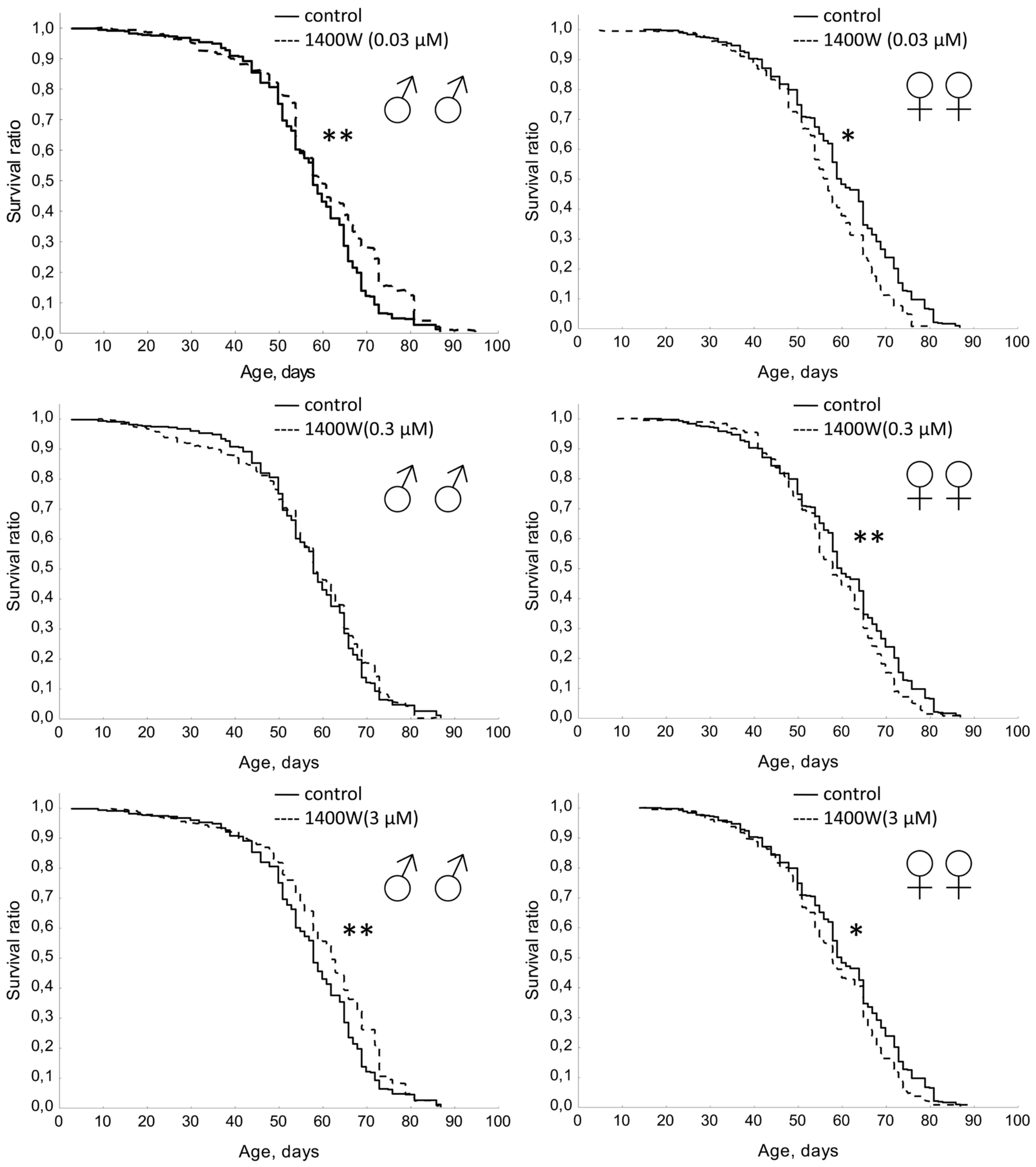

Figure S11: Effect of 1400W (0.03, 0.3, 3 µM) on lifespan Drosophila melanogaster. * $p< 0.001$, ** $p< 0.05$ (Kolmogorov-Smirnov test).

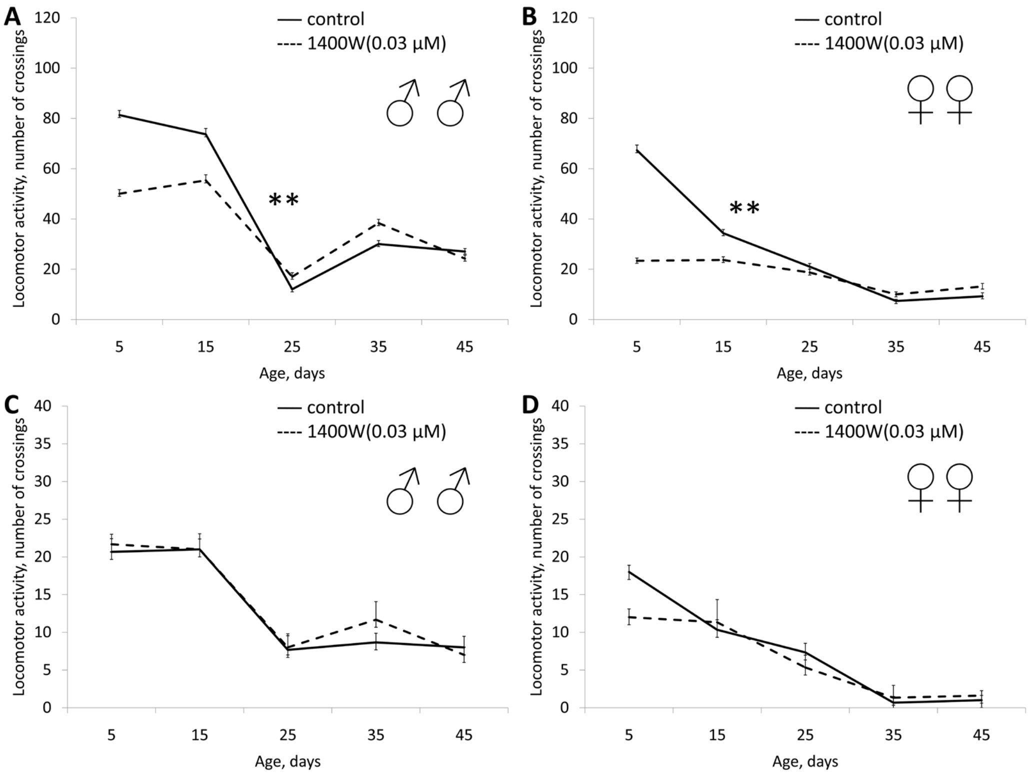

Figure S12: Effect of 1400W (0.03 µM) on locomotor activity Drosophila melanogaster. (A, B) spontaneous activity. (C, D) negative geotaksis test. * $p< 0.001$, ** $p< 0.05$ (χ^2 test).

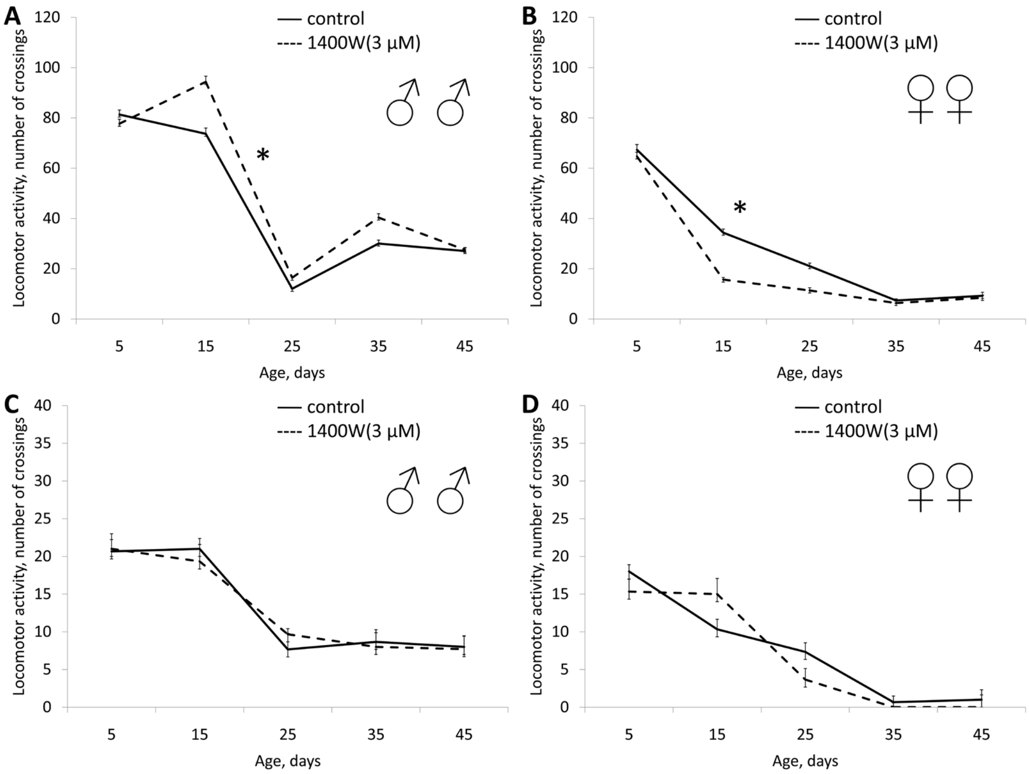

Figure S13: Effect of 1400W (3 µM) on locomotor activity Drosophila melanogaster. (A, B) spontaneous activity. (C, D) negative geotaksis test. * $p< 0.001$, ** $p< 0.05$ (χ^2 test).

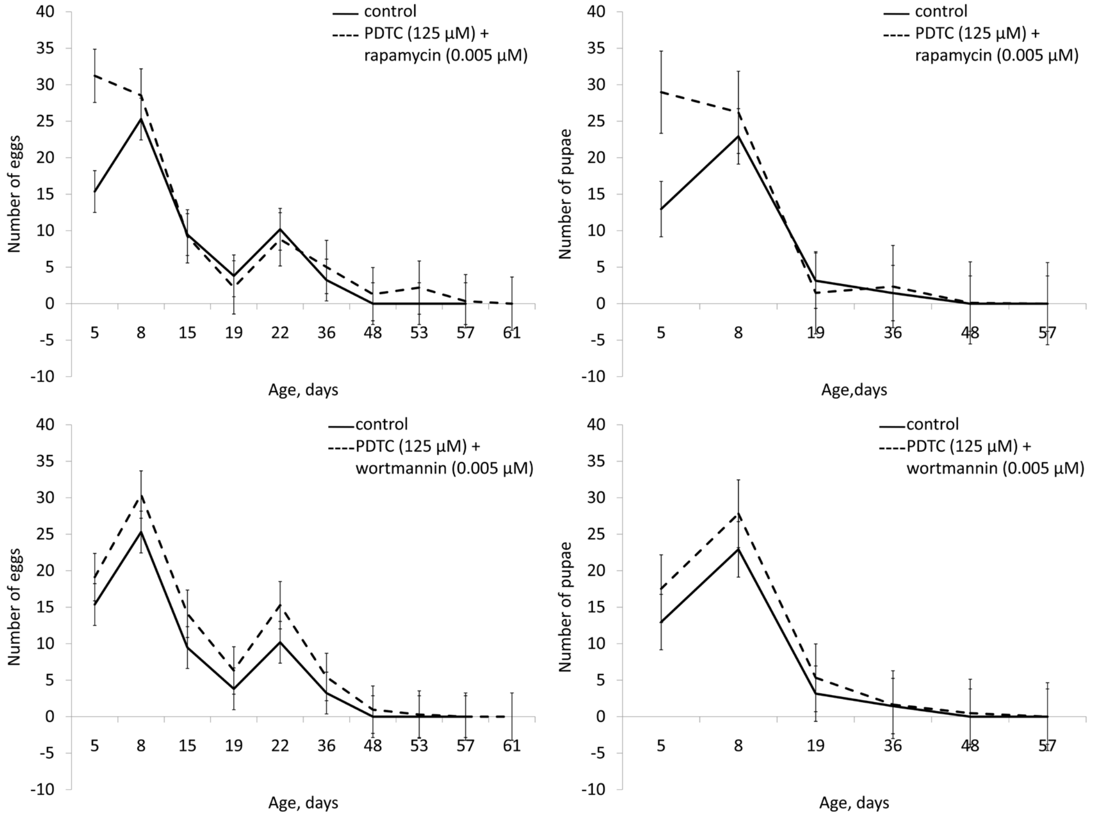

Figure S14: Effect of combined use of PDTC (125 µM) and rapamycin (0.005 µM), mix PDTC (125 µM) and wortmannin (0.005 µM) on fertility of females Drosophila melanogaster. * $p< 0.001$, ** $p< 0.05$ (χ^2 test).
